# Urinary dysfunction in patients with rectal cancer: a prospective cohort study

**DOI:** 10.1111/codi.14784

**Published:** 2019-08-07

**Authors:** L. Karlsson, D. Bock, D. Asplund, B. Ohlsson, J. Rosenberg, E. Angenete

**Affiliations:** ^1^ Department of Surgery Institute of Clinical Sciences SSORG – Scandinavian Surgical Outcomes Research Group Sahlgrenska Academy at University of Gothenburg Gothenburg Sweden; ^2^ Region Västra Götaland Sahlgrenska University Hospital/Östra Department of Surgery Gothenburg Sweden; ^3^ Department of Surgery Blekinge Hospital Karlshamn Sweden; ^4^ Department of Surgery Herlev Hospital University of Copenhagen Herlev Denmark

**Keywords:** Urinary dysfunction, rectal cancer, PROM

## Abstract

**Aim:**

Urinary dysfunction is one of many complications after treatment for rectal cancer. The aim of this study was to evaluate the prevalence of patient‐reported urinary dysfunction at the time of diagnosis and at 1‐year follow‐up and to assess the risk factors linked to urinary incontinence.

**Method:**

Patients with newly diagnosed rectal cancer were included in the QoLiRECT study between 2012 and 2015. Questionnaires from the time of diagnosis and 1‐year follow‐up were analysed, with 1085 and 916 patients, respectively, eligible for analysis. Regression analyses were made to investigate possible risk factors for incontinence. The patient cohort was also compared with a cohort from the Swedish general population.

**Results:**

At baseline, the prevalence of urinary dysfunction (14% of women, 8% of men) was similar to that in the general population. At 1‐year follow‐up, 20% of patients experienced urinary incontinence (29% of women, 14% of men). Emptying difficulties were experienced by 46% (41% of women, 49% of men) and urgency by 58% across both sexes. Abdominoperineal excision and urinary dysfunction at baseline were found to be independent risk factors for incontinence at 1‐year follow‐up. Among patients who were continent at baseline, risk factors were female sex, physical inactivity at baseline, comorbidity and abdominoperineal excision.

**Conclusion:**

Urinary dysfunction is frequent among patients with rectal cancer, with up to a two‐fold increase in symptoms 1 year after diagnosis. Unfortunately, few factors are modifiable and these results stress the importance of informing patients of possible outcomes related to urinary dysfunction after treatment for rectal cancer.

**What does this paper add to the literature?**

It is important to improve our knowledge about possible side effects affecting patients after treatment for rectal cancer. This article shows that urinary dysfunction is common in a large unselected population of patients with rectal cancer, with risk factors for incontinence related to both the patient and the cancer treatment.

## Introduction

Rectal cancer is one of the most common cancers in the world. Survival has improved over the last decades 1, mostly due to improved treatment 2. However, treatments may cause morbidity and various forms of functional impairment 3. With improved survival, functional impairment as reported by the patient has received increasing attention. One such impairment is urinary dysfunction.

Previous studies have shown a prevalence of urinary dysfunction of between 6% and 77% after rectal cancer surgery 4, 5. Different definitions, along with different time frames, may explain this variation in the reported prevalence of urinary dysfunction. There is no general consensus in the literature about how to define urinary dysfunction after rectal cancer surgery. Many different scores and descriptions have previously been used, such as the International Prostate Symptom Score (IPSS) 6, 7, 8, 9, long‐term catheterization 10, voiding problems 11, 12 or urinary incontinence 13. The studied time frame varies from 7 days 14 up to 5 years 13 after surgery.

The underlying mechanism in the development of urinary dysfunction in patients treated for rectal cancer is not completely clear. There are conflicting views on, for example, the impact of preoperative radiotherapy 5, 13, 15, age 15, 16, sex 13, 17 and type of surgery 5, 7, 15, 18 on urinary dysfunction. Most studies recognize that nerve damage during surgery and preoperative urinary dysfunction are important risk factors 10, 13, 19.

The aim of this study was to investigate the prevalence of urinary dysfunction in patients with rectal cancer prior to treatment and at 1‐year follow‐up. A secondary objective was to identify possible preoperative risk factors for the development of urinary incontinence at 1‐year follow‐up.

## Method

### The QoLiRECT study

Quality of Life in Rectal Cancer (QoLiRECT) is a prospective, observational multicentre study of patients with rectal cancer. The study included patients from 16 Swedish and Danish hospitals. All patients above the age of 18 years with a confirmed adenocarcinoma of the rectum, i.e. up to 15 cm from the anal verge, who understood either Swedish or Danish were eligible for inclusion. Patients were asked to participate in the study at the time of diagnosis but before initiation of treatment. Those who gave informed consent were asked to complete a detailed questionnaire. Between February 2012 and September 2015, 1248 patients were included in the study. A total of 1085 patients returned the questionnaire at baseline. Patients were subsequently contacted by the study secretariat and asked to fill out similar questionnaires at 1, 2 and 5 years after the diagnosis. All patients included in the study were contacted at all follow‐up points, regardless of whether they had filled out the baseline questionnaire or not. A total of 920 patients answered the 1‐year questionnaire, including 33 patients who had not answered the baseline questionnaire. This analysis is based on data from the questionnaire at baseline and at 1‐year follow‐up. Cystectomy at index rectal cancer surgery was an exclusion criterion for 1‐year follow‐up analyses, rendering 916 patients eligible for analysis.

### Questionnaire data

The questionnaires included items covering socioeconomic background, lifestyle factors, overall quality of life as well as urinary, bowel, sexual and stoma function. The questionnaires were developed using validated and previously described methods 20, 21. In‐depth qualitative interviews with content analysis to derive new questions were used together with previously validated questions 21, 22. Content validation was performed by an expert panel. The questionnaires were face‐to‐face validated by patients with rectal cancer 20, 21, 23. Patients were asked about the quality, frequency and intensity of symptoms as well as the corresponding distress with a recall period of 1 month 21.

In this article, the questions regarding urinary function were analysed. The baseline and follow‐up questionnaires included 12 questions on urinary function 21, 24, 25.

During analysis, the 12 questions were divided into four categories: incontinence, bladder emptying difficulties, urgency and distress. Incontinence was defined as ‘incontinence during daytime and/or night once per week or more’. Both bladder emptying difficulties and urgency were divided into ‘never’, ‘up to 50% of the time’ and ‘more than 50% of the time’. The symptom‐associated distress was derived from the question ‘If your urinary function would remain as it is now for the rest of your life, how would you feel about that?’ with a cut‐off between ‘It would not bother me at all’ and ‘It would somewhat bother me’.

Questions on smoking, alcohol use and physical activity were also analysed. Patients were regarded as smokers if they answered ‘Yes, I smoke’ regardless of the amount per week. Former and never smokers were merged into one group. Alcohol use was dichotomized between drinking less than or more than 16 standard glasses per week, as has been described in a previous study 26. Physical activity was measured using the Saltin–Grimby Physical Activity Level Scale 27 with a cut‐off between ‘Physically inactive’ and ‘Some light physical activity, at least 4 h per week’. Comorbidity was analysed from a question on the presence of a number of health conditions, including renal disorders, cardiovascular disorders and diabetes, among others. It was defined as the presence of one or more of these conditions.

### Swedish reference population

A reference sample of 3000 people born between 1924 and 1983 was derived from the Swedish tax agency. They were contacted between June 2014 and November 2015 and asked to fill out a questionnaire about their health‐related quality of life. A total of 1078 people (median age 63 years, range 31–90, 53% women) completed and returned the questionnaire. Eight questions related to urinary function corresponded to the questions in the QoLiRECT questionnaires. This allowed for comparison between the QoLiRECT population at baseline and 1‐year follow‐up and the Swedish reference population.

### National registries

Data on sex, age, American Society of Anesthesiologists (ASA) classification, tumour stage according to the Union for International Cancer Control (UICC) classification, chemo‐ and/or radiotherapy, body mass index (BMI), type of surgery (anterior resection or abdominoperineal excision), open or laparoscopic technique and perioperative blood loss were derived from the Swedish ColoRectal Cancer Registry (SCRCR) and from the Danish Colorectal Cancer Group (DCCG) registry. Because of the lack of some of surgical and oncological data in the DCCG registry, a clinical record form was developed at the study secretariat and filled out retrospectively by personnel at the participating Danish hospitals. Data regarding pre‐ and postoperative chemotherapy, preoperative radiotherapy and type of surgery in the Danish patient population were derived from this clinical record form. Because of frequent missing data in the SCRCR regarding postoperative chemotherapy, this information was retrieved from the 1‐year follow‐up questionnaire when it was missing in the registry.

### Statistical analysis

Prior to analysis, a plan for statistical analysis was developed and finalized. The prevalence of bladder emptying difficulties (more than 50%), day and/or night‐time urinary incontinence (at least once every week) and urinary urgency (more than 50%) were compared with the reference population separately for men and women in an age‐adjusted analysis using the modified Poisson regression approach of Zou 28.

In the analysis of potential risk factors for urinary incontinence during day and/or night‐time at 1 year the following variables were considered: incontinence at baseline, gender, age, smoking status, alcohol consumption, physical activity, comorbidity (baseline variables) and ASA classification, type of surgery (abdominoperineal excision or any other surgery performed such as anterior resection with or without an anastomosis, i.e. Hartmann), technique (laparoscopy versus open), bleeding during surgery, tumour stage (UICC), preoperative chemoradiotherapy, radiation therapy and postoperative chemotherapy (intra‐operative and 1‐year follow‐up variables).

It was hypothesized that preoperative incontinence was a descendant of the baseline variables on causal paths and exploratory analysis confirmed that age, gender, smoking status and comorbidity were associated with preoperative incontinence. This was also confirmed in an analysis of the reference population. It was further found that incontinence at baseline and at 1 year was highly correlated. Consequently, in the analysis of incontinence at 1 year, evaluation was restricted to intra‐operative and 1‐year follow‐up variables, and baseline information was assessed solely through preoperative incontinence. In a subgroup of preoperatively continent patients, both baseline, intra‐operative and 1‐year follow‐up variables were included in the risk factor assessment.

The variables were evaluated using simple and bivariate logistic regression analysis (urinary continence before treatment being included as a fixed effect). A subset of variables was selected using the purposeful selection strategy for logistic regression with the SAS macro 29. Missing values in the variables selected from the strategy were imputed by multiple imputations by chained equations 30. The final modelling involved the subsequent estimation and pooling of the multiple regression model across the imputations using proc mianalyze in sas. spss version 25 (IBM Corp., Armonk, New York, USA), sas version 9.4 (SAS Institute, Cary, North Carolina, USA) and r version 3.4.3 (R Foundation for Statistical Computing, Vienna, Austria) were used for calculations and graphics.

### Ethics

The QoLiRECT study has obtained ethical approval from the Central Ethical Review Board in Sweden (Dnr 595‐11), the Danish Data Protection Agency (HEH.750.89‐21, HGH‐2016‐016) and the Regional Ethical Review Board in Denmark (H‐3‐2012‐FSP26).

## Results

**Figure 1:**
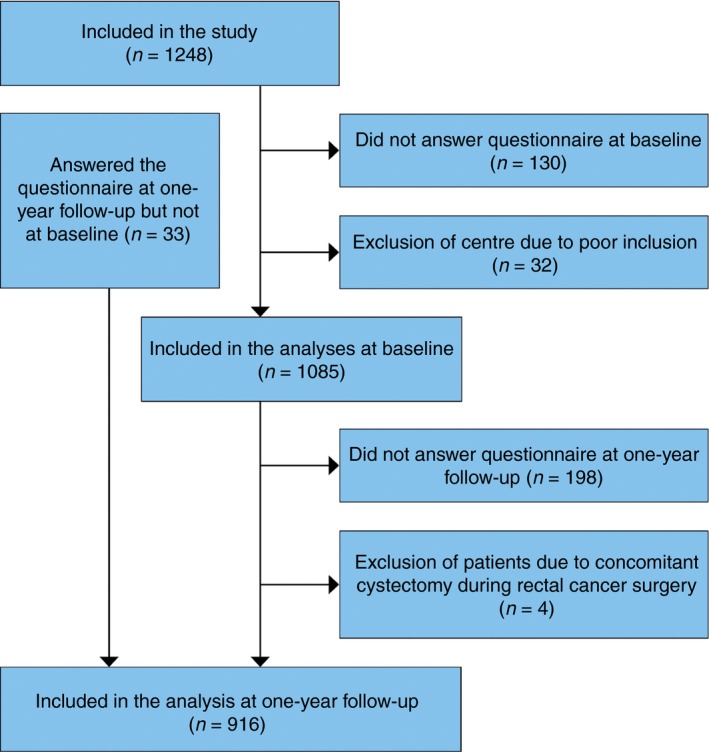
Flow chart.

Altogether 1085 and 920 patients were available for analysis at baseline and at 1‐year follow‐up, respectively (Fig. 1). Four patients were excluded at 1 year due to a concomitant cystectomy at their index surgery. One centre was excluded due to a poor inclusion rate in relation to their patient population, giving low external validity. The cohort comprised 64% men. Women had a lower ASA classification and less blood loss than men. Otherwise, there were no clinically relevant differences between men and women regarding comorbidity, lifestyle factors or treatment (Table 1). Patients lost to follow‐up between baseline and the 1‐year follow‐up are presented in Table 2, indicating no systematic bias in the dropout.

**Table 1:** Demography at baseline.

	Women (*n* = 392), (%)	Men (*n* = 693), (%)	Total (*n* = 1085), (%)	Missing data
Curative intended treatment	375 (96)	637 (92)	1012 (93)	0
Palliative intended treatment	17 (4)	56 (8)	73 (7)	0
Age (years), median (range)	68 (25–93)	69 (38–100)	69 (25–100)	0
BMI (kg/m^2^), median (range)	25 (22–28)	26 (24–28.5)	25.5 (23–28.5)	162
ASA classification				
I	96 (27)	139 (23)	235 (25)	133
II	213 (61)	351 (58)	564 (59)	
III–IV	42 (12)	111 (18)	153 (16)	
Comorbidity				
Yes	234 (60)	427 (62)	661 (62)	12
No	153 (40)	259 (38)	412 (38)	
Current smoker				
Yes	34 (9)	65 (10)	99 (9)	20
No	353 (91)	613 (90)	966 (91)	
Alcohol intake				
More than 16 glasses per week	4 (1)	34 (5)	38 (4)	46
Less than 16 glasses per week	370 (99)	631 (95)	1001 (96)	
Physical activity				
Physically inactive	54 (14)	102 (15)	156 (15)	48
Some light physical activity or more	324 (86)	557 (85)	881 (85)	
UICC classification*				
0	6 (2)	12 (2)	18 (2)	140
I	114 (33)	164 (27)	278 (29)	
II	80 (23)	144 (24)	224 (24)	
III	110 (32)	186 (31)	296 (31)	
IV	35 (10)	94 (16)	129 (14)	
Preoperative radiation therapy				
Yes	152 (39)	228 (33)	380 (35)	0
No	240 (61)	465 (67)	705 (65)	
Preoperative chemoradiotherapy				
Yes	63 (16)	154 (22)	217 (20)	0
No	329 (84)	539 (78)	868 (80)	
Postoperative chemotherapy				
Yes	145 (39)	246 (38)	391 (38)	64
No	226 (61)	404 (62)	630 (62)	
Operative technique				
Open	162 (46)	299 (50)	461 (48)	133
Laparoscopic	190 (54)	301 (50)	491 (52)	
Type of surgery				
Anterior resection	209 (56)	293 (45)	502 (49)	56
Abdominoperineal excision	108 (29)	223 (34)	331 (32)	
Hartmann's procedure	24 (6)	64 (10)	88 (9)	
No intervention	19 (5)	48 (7)	67 (7)	
Other†	15 (4)	26 (4)	41 (4)	
Perioperative blood loss (ml), (range)	150 (50–300)	250 (100–500)	200 (50–450)	166

*UICC is based on pTNM classification.

†Other includes colectomy, transanal endoscopic microsurgery, local excision, laparotomy without excision and unknown.

**Table 2:** Description of patients who dropped out between baseline and 1‐year follow‐up.

	Total population at baseline (*n* = 1085)	Missing information	Drop‐out between baseline and 1 year follow‐up (*n* = 198)	Missing information at drop‐out
Age (years)	69 (25–100)		71 (35–93)	
Sex				
Female	392 (36)		60 (30)	
Male	693 (64)		138 (70)	
Deceased at 1‐year follow‐up	0		49	
ASA classification				
I	235 (25)	133	30 (21)	52
II	564 (59)		85 (58)	
III–IV	153 (16)		31 (21)	
Type of surgery				
Anterior resection	502 (49)	56	62 (34)	16
Abdominoperineal resection	331 (32)		49 (27)	
Hartmann's procedure	88 (9)		25 (14)	
No intervention	67 (7)		37 (20)	
Other*	41 (4)		9 (5)	
UICC classification†				
0	18 (2)	140	3 (2)	41
I	278 (29)		33 (21)	
II	224 (24)		37 (24)	
III	296 (31)		50 (32)	
IV	129 (14)		34 (22)	

*Other includes colectomy, transanal endoscopic microsurgery, local excision, laparotomy without excision and unknown.

†UICC is based on pTNM classification.

Urinary incontinence was more common among women than men at both baseline (14% *vs* 8%) and 1‐year follow‐up (29% *vs* 14%) (Table 3). Both men and women had a similar pattern, with almost a two‐fold increase in incontinence 1 year after diagnosis.

**Table 3:** Prevalence of urinary dysfunction during the last month.

	Female population at baseline (*n* = 392), (%)	Female population at 1‐year follow‐up (*n* = 347), (%)	Male population at baseline (*n* = 693), (%)	Male population at 1‐year follow‐up (*n* = 569), (%)	Total population at baseline (*n* = 1085), (%)	Total population at 1‐year follow‐up (*n* = 916), (%)
Urinary incontinence during daytime and/or nighttime						
At least once per week	52 (14)	100 (29)	53 (8)	81 (14)	105 (10)	181 (20)
Less than once per week	329 (86)	239 (71)	624 (92)	478 (86)	953 (90)	717 (80)
Urinary incontinence during daytime						
At least once per week	41 (11)	98 (29)	45 (7)	79 (14)	86 (8)	177 (20)
Less than once per week	340 (89)	241 (71)	638 (93)	480 (86)	978 (92)	721 (80)
Urinary incontinence during the night						
At least once per week	26 (7)	33 (10)	20 (3)	26 (5)	46 (4)	59 (7)
Less than once per week	361 (93)	305 (90)	657 (97)	533 (95)	1018 (96)	838 (93)
Urinary incontinence only during physical activity						
Yes	100 (26)	129 (39)	22 (3)	48 (9)	122 (11)	177 (20)
No	65 (17)	85 (25)	106 (16)	137 (25)	171 (16)	222 (25)
Not applicable	216 (57)	120 (36)	554 (81)	372 (67)	770 (72)	492 (55)
Urinary incontinence during sexual activity						
Yes	4 (1)	5 (1)	10 (1)	3 (1)	14 (1)	8 (1)
No	91 (24)	71 (21)	207 (31)	135 (24)	298 (28)	206 (23)
Not applicable	287 (75)	258 (77)	458 (68)	417 (75)	745 (70)	675 (76)
Use of pads or other aids for urinary incontinence						
Yes	79 (20)	132 (39)	35 (5)	62 (11)	114 (11)	194 (22)
No	78 (20)	50 (15)	146 (22)	117 (21)	224 (21)	167 (19)
Not applicable	229 (59)	155 (46)	489 (73)	370 (67)	718 (68)	525 (60)
Bladder emptying difficulties						
More than 50% of the time	23 (6)	37 (11)	46 (7)	61 (11)	69 (6)	98 (11)
Up to 50% of the time	84 (22)	103 (30)	247 (37)	208 (38)	104 (10)	311 (35)
Never or not applicable	274 (72)	199 (59)	383 (57)	280 (51)	884 (83)	479 (54)
Use of aids for bladder emptying (catheter or others)						
Yes	3 (1)	12 (4)	18 (3)	59 (10)	21 (2)	71 (8)
No	383 (99)	325 (96)	664 (97)	504 (90)	1047 (98)	829 (92)
Urgency to urinate						
More than 50% of the time	18 (5)	31 (9)	23 (3)	41 (7)	41 (4)	72 (8)
Up to 50% of the time	137 (36)	166 (49)	254 (38)	280 (51)	391 (37)	446 (50)
Never	196 (51)	129 (38)	326 (49)	192 (35)	522 (50)	321 (36)
Not applicable	33 (9)	14 (4)	67 (10)	41 (7)	100 (9)	55 (6)
Wet themselves because of inability to reach the toilet in time						
More than 50% of the time	5 (1)	3 (1)	3 (0)	4 (1)	8 (1)	7 (1)
Up to 50% of the time	51 (13)	31 (9)	41 (6)	17 (3)	92 (9)	48 (5)
Never or not applicable	329 (85)	307 (90)	624 (93)	531 (96)	953 (91)	838 (94)
If your urinary function would remain as it is now for the rest of your life, how would you feel about that?						
Distressed	75 (20)	125 (37)	112 (17)	174 (32)	187 (18)	299 (34)
Not distressed	35 (9)	40 (12)	51 (8)	50 (9)	86 (8)	90 (10)
Not applicable	270 (71)	174 (51)	510 (76)	328 (59)	780 (74)	502 (56)

Bladder emptying difficulties were prevalent to a higher extent in men if all problems were included (43% at baseline and 49% at 1 year) but a more pronounced increase was seen in women at 1 year (28% at baseline and 41% at 1 year) (see Table 3). More men than women used aids for bladder emptying at 1‐year follow‐up.

The prevalence of urgency was similar in men and women, and increased by approximately 40% at 1 year.

Almost 20% of all patients, both men and women, were distressed about their urinary symptoms at baseline. At 1 year, this figure almost doubled.

**Figure 2:**
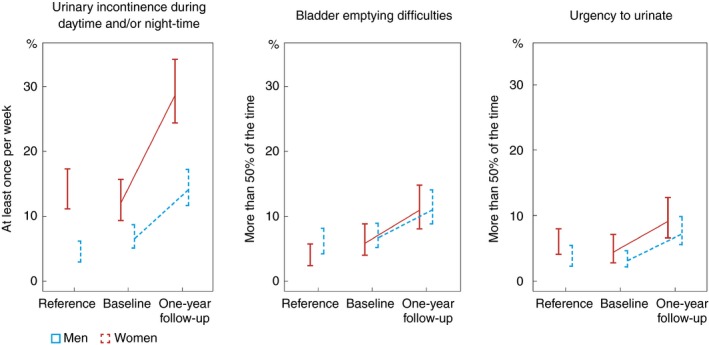
Comparison of study population with Swedish reference population.

Patients reported similar urinary dysfunction to the Swedish reference population at baseline (Fig. 2). At 1 year, patient‐reported dysfunction increased in both men and women, as described above.

**Figure 3:**
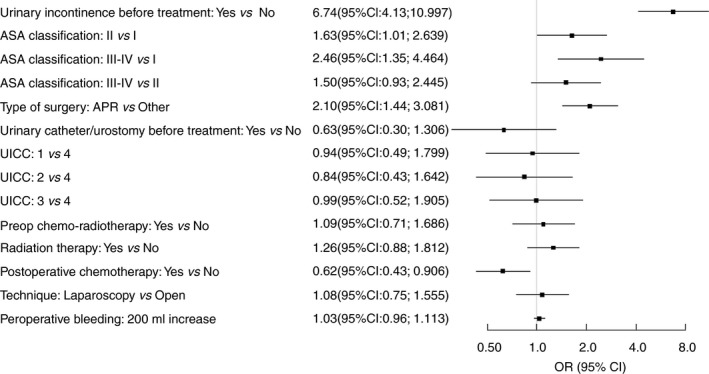
Intra‐operative and one‐year follow‐up risk factors for incontinence (bivariate analysis with urinary continence before treatment included as a fixed effect). Presented as odds ratio and 95% confidence intervals.

**Figure 4:**
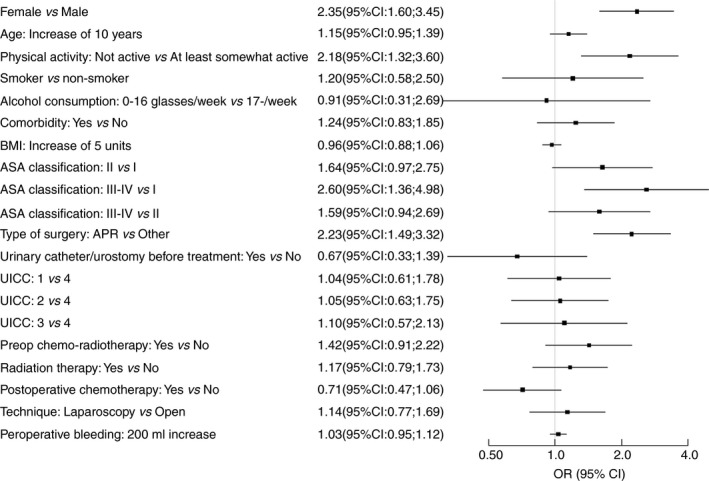
Subgroup of preoperatively continent patients. Baseline, intra‐operative and one‐year follow‐up risk factors for incontinence (univariate analysis). Presented as odds ratio and 95% confidence intervals.

Risk factors for urinary incontinence are shown in Fig. 3. Preoperative incontinence as well as abdominoperineal excision were independent risk factors for incontinence at 1‐year follow‐up (Table 4). Comparing abdominoperineal excision and anterior resection, we found that all urinary problems were more common after abdominoperineal excision [urinary incontinence (27% *vs* 17%, *P* < 0.05), urgency (12% *vs* 5%, *P* < 0.05) and evacuation problems (14% *vs* 9%, *P* < 0.05)]. The differences regarding surgical technique were more pronounced in women with regard to urinary incontinence and urgency, but evacuation problems were a little more pronounced in men who underwent abdominoperineal excision. Risk factors for incontinence in the subgroup of preoperatively continent patients at 1‐year follow‐up are shown in Fig. 4. Identified risk factors were female sex, physical inactivity at baseline, higher ASA classification and abdominoperineal excision.

**Table 4:** Multiple regression with variables chosen from the variable selection strategy.*

Variable	Comparison	OR (95% CI)	*P*‐value
All evaluable patients at 1‐year follow‐up (*n* = 916)			
Urinary incontinence before treatment	Yes *vs* No	6.61 (4.01; 10.87)	< 0.001
Surgical method	APE *vs* other	2.02 (1.38; 2.94)	< 0.001
Subgroup: preoperatively continent patients (*n* = 782)			
Sex	Female *vs* male	2.57 (1.73; 3.84)	< 0.001
Physical activity	Not active *vs* at least somewhat active	1.88 (1.07; 3.31)	0.0279
ASA classification	II *vs* I	1.68 (0.98; 2.88)	0.0574
	III–IV *vs* I	2.66 (1.34; 5.29)	0.005
	III–IV *vs* II	1.58 (0.90; 2.78)	0.1089
Surgical method	APE *vs* other	2.15 (1.42; 3.27)	< 0.001
Surgical technique	Laparoscopic *vs* Open	1.26 (0.84; 1.90)	0.2621

APE, abdominoperineal excision.

*Multiple imputations by chained equations with 50 imputations was used for the selected variables.

## Discussion and conclusion

The present study showed that urinary dysfunction is a frequent problem from which many patients suffer a long time after they have received their rectal cancer diagnosis and treatment.

Of the various components of urinary dysfunction, urinary incontinence appears to be the most important problem. The study indicates that incontinence shows a two‐fold increase in the patient population following treatment, with approximately one‐third of patients experiencing incontinence at least once weekly. As expected, women had more problems than men (29% compared with 14%), although the increase in incontinence was similar between sexes. There are no data in our study that can explain these differences with regard to tumour stage or surgical technique as this was fairly similar between sexes.

Risk factors for urinary incontinence were incontinence at baseline and abdominoperineal excision. Among patients who were continent at baseline, risk factors were female sex, physical inactivity at baseline, higher ASA classification and abdominoperineal excision. Apart from physical inactivity, these risk factors have been reported previously 5, 7, 13, 15, 17, 31, 32. In the present study, neither chemotherapy nor radiotherapy was found to be a significant risk factor, which is also consistent with previous findings 13.

We identified the modifiable risk factor of physical inactivity in our study. This warrants further research on how to increase physical activity before treatment in order to improve recovery and possibly urinary function. The question arises as to whether physical therapy before and after treatment could reduce the number of patients with urinary incontinence.

A previous study in 785 patients reported that 38% of patients experienced incontinence and 31% had bladder emptying difficulties 5 years after surgery 13. It is probable that the differences in the time frame and the type and interpretation of the questionnaires may explain the different results. However, their 1‐year data indicate 30% urinary incontinence and bladder emptying difficulties, which is similar to the present study 13.

In a study on 516 women who underwent abdominoperineal excision or low anterior excision in Denmark between 2001 and 2007, it was reported that 63% and 77% of patients reported incontinence and urgency, respectively 5. That study used the ICIQ‐FLUTS questionnaire to evaluate urinary dysfunction 33. Although the questions are similar to the present study, the responses were dichotomized into ‘occasionally/sometimes/most of the time/all the time’ or ‘never’ and then further dichotomized according to median scores. This dichotomization could be an explanation for the differences between their results and ours. The present study cohort also includes more surgical alternatives, including no surgery at all, and we studied a shorter time frame.

In the AbdominoPerineal Extralevator Resection study (APER), a Swedish cross‐sectional study in patients who underwent abdominoperineal excision between 2007 and 2009, 46.5% of patients were regarded as incontinent 32. However, the definition of incontinence was different from the present study and all patients underwent abdominoperineal excision.

The fact that there is a near two‐fold increase in distress in terms of urinary dysfunction over time clearly indicates that such problems are important to patients and stresses the need to try to prevent and alleviate posttreatment urinary dysfunction. This is of even greater importance when taking into account the fact that one of the risk factors is modifiable.

The main strength of this study is its prospective, unselected, multicentre design and that all treatment possibilities are represented, both palliative and curative. As has been previously reported 34, the patient population is representative of the total Swedish and Danish rectal cancer population. The cohort size is large, with good response rates at baseline and at 1‐year follow‐up. The questionnaires were developed using previously validated questions. The use of a reference population with similar results at baseline is also a strength and indicates that patients’ problems with urinary dysfunction at 1‐year follow‐up are largely related to their treatment.

There are some limitations to this study. One is the observational design, which makes it difficult to draw conclusions about causation for the main results. Another limitation is the fact that only patients who had a good understanding of either Swedish or Danish could participate. However, as previously reported, results have shown the present cohort to be representative, indicating that this is only a minor limitation 34.

As the results indicate that a substantial number of all rectal cancer patients will suffer long‐term urinary dysfunction, we stress the importance of informing patients of this prior to treatment. In a previous study in the QoLiRECT population 35, only 32% of patients reported that they had received information about the possible effects of treatment on urinary function. Since abdominoperineal excision was the only surgical risk factor for incontinence, it is of even greater importance to inform these patients about the possible long‐term dysfunctional outcomes.

In conclusion, many patients treated for rectal cancer develop urinary dysfunction, with substantial distress after treatment. Incontinence appears to be the most relevant problem, with a two‐fold increase compared with baseline levels. It is important to inform patients of possible treatment‐related functional impairments, to identify patients who are at particular risk and to ensure a proper posttreatment follow‐up. Further evaluation of modifiable lifestyle factors is warranted to possibly prevent morbidity after treatment for rectal cancer.

## Conflicts of interest

None of the authors have any conflicts of interest.
